# CD73 expression and clinical significance in human metastatic melanoma

**DOI:** 10.18632/oncotarget.25426

**Published:** 2018-06-01

**Authors:** Inês Monteiro, Selena Vigano, Mohamed Faouzi, Isabelle Treilleux, Olivier Michielin, Christine Ménétrier-Caux, Christophe Caux, Pedro Romero, Laurence de Leval

**Affiliations:** ^1^ Institute of Pathology, Lausanne University Hospital, Lausanne, Switzerland; ^2^ Department of Oncology, Faculty of Biology and Medicine, University of Lausanne, Lausanne, Switzerland; ^3^ Institute of Social and Preventive Medicine (IUMSP), University Hospital, Lausanne, Switzerland; ^4^ Anatomopathology Department, Centre Léon Bérard, Lyon, France; ^5^ Department of Oncology, Lausanne University Hospital, Lausanne, Switzerland; ^6^ Université Claude Bernard Lyon 1, Centre de recherche en cancérologie de Lyon, Lyon, France; ^7^ Department of Innovation and Translational Research, Centre Léon Bérard, Lyon, France

**Keywords:** CD73, ecto-5'-nucleotidase, immunohistochemistry, melanoma, prognosis

## Abstract

**Background:**

CD73 is an ectoenzyme involved in the production of adenosine. It exerts immunosuppressive and protumoral roles and has emerged as a potential immuno-oncology target.

**Results:**

CD73 expression was detected in TC in 54% of melanoma metastases, involving < 50% TC in the majority of the cases, with variable intensity. CD73 expression was significantly associated with a lower Breslow's depth of the primary lesion and was more frequent in patients having received prior non-surgical therapies. In an adjusted analysis, CD73 expression in TC (H-score > 37.5 or intensity > 1) significantly correlated to decreased overall survival (OS) from biopsy. Of the samples containing TIMC, 35% presented CD73+ TIMC. Highly infiltrated tumors were more likely to contain CD73+ TIMC. CD73 expression in TIMC (percentage ≥1%) significantly correlated with improved OS from biopsy.

**Conclusions:**

Immunohistochemistry detected CD73 expression in more than half of metastatic melanomas. While CD73 expression in TC significantly correlated with decreased OS, CD73 expression in TIMC significantly associated with improved OS. These results encourage the study of anti-CD73 therapies for metastatic melanoma patients.

**Methods:**

CD73 expression was assessed by immunohistochemistry in metastatic melanomas from 114 patients. Immunostainings were evaluated in tumor cells (TC) (percentage, intensity (1–3) and H-score) and in tumor-infiltrating mononuclear cells (TIMC) (percentage).

## INTRODUCTION

CD73 is an ectoenzyme with a 5′-nucleotidase activity, which catalyzes the rate-limiting step in the generation of extracellular adenosine [1–3]. In addition, CD73 has an adhesion role participating in cell-cell and cell-matrix interactions [1, 4–6]. Structurally, CD73 consists of two identical 70-kD subunits, each with an N-terminal domain (containing binding sites for catalytic ions) and a C-terminal domain (containing an AMP binding site), anchored to the plasma membrane [1]. In physiological conditions, CD73 is expressed by stromal cells, follicular dendritic cells, and endothelial cells [1] but also by variable proportions of adaptive immune cells (B cells and some T-cell subsets) [7, 8]. CD73 expression and function is increased by hypoxic conditions and several inflammatory mediators [1].

CD73 participates in the catabolism of extracellular ATP which is first converted by the ectoenzyme CD39 to ADP and AMP, the latter being transformed to adenosine by CD73 [3]. In conditions of ischemia, hypoxia or inflammation, extracellular adenosine levels increase [1]. In these circumstances, adenosine down-regulates inflammatory and immune responses, modulating the amplitude of physiological responses and preventing collateral tissue damage [3]. Adenosine promotes regulatory T cell function, decreases T helper 1 and natural killer cell activity, inhibits M1 macrophage activation, promotes macrophage M2 differentiation and drives dendritic cells towards an anti-inflammatory cytokine profile [3, 9]. The four transmembrane adenosine receptors (A_1_, A_2a_, A_2b_ and A_3_) are expressed by immune (mostly A_2a_, A_2b_) and endothelial cells [3].

Adenosine stimulates the production of vascular endothelial growth factor by endothelial cells and macrophages, inducing angiogenesis [3]. When expressed by vascular endothelial cells, CD73 produces adenosine, which, by a paracrine effect, inhibits vascular permeability and lymphocyte trafficking [10]. Conversely, CD73 engagement on human peripheral blood lymphocytes increases their binding to endothelial cells by increasing LFA-1 avidity in a non-enzymatic way [6].

The adenosine cycle can be viewed as a metabolic immune checkpoint, and strategies to interfere with this cascade seem promising [1].

Several immunohistochemistry (IHC)-based studies of human samples have been recently published. In colorectal cancer, higher tumoral CD73 and lower stromal CD73 expression significantly associated with higher TNM stage, presence of lymphatic metastasis and poor tumor differentiation. Expression in tumor cells also associated with a higher risk of death [11]. CD73 expression on tumor cells of triple negative breast cancer was significantly associated with reduced overall survival and negatively correlated with tumor immune infiltration [12]. In primary head and neck squamous cell carcinomas and corresponding metastatic lymph nodes, CD73 expression correlated positively with tumor stage and associated with reduced overall survival [13]. Papillary thyroid and pancreatic ductal carcinomas present higher CD73 expression compared with normal thyroid or pancreas tissue, respectively [14, 15].

In ovarian carcinoma, CD73 overexpression associated with better 5-year overall survival. This may be due to that overexpression of CD73 was more frequently observed in mucinous and clear cell adenocarcinomas compared to serous or endometrioid adenocarcinomas and in patients with known good prognostic factors. The CD73-negative group presented significantly more infiltration of regulatory T cells [16].

Regarding melanoma, one recent study showed that cell lines derived from metastatic melanomas express more CD73 than those derived from normal melanocytes or primary melanomas [5]. Primary melanomas that do not epigenetically downregulate the transcription of the NT5E gene (that encodes CD73) were found to metastasize more often [17]. Studying melanoma cells in CD73-deficient mice, CD73 was shown to promote MAP-kinase signaling, tumor growth and angiogenesis [18]. The same study reported tumor cell-associated CD73 contribution to metastasis formation through attachment to endothelium [18]. In addition, the CD73-tenascin-C complex was shown to be involved in cell migration and invasion in melanoma cell lines [4, 5]. In a mouse model of melanoma, the use of a specific CD73 inhibitor improved T- and B-cell-mediated anti-tumor immunity and reduced tumor growth [19].

In this context, our study aims at characterizing CD73 expression in human metastatic melanoma, its association with clinicopathological parameters and its prognostic impact.

## RESULTS

### Clinicopathological features

The demographics and clinicopathological features are summarized in Table 1. Patients comprised 55 men and 59 women with a median age of 67 years at the time the examined metastatic biopsy was excised (range 23–91 years). The corresponding primary melanomas were cutaneous in the majority of cases (79%) while ocular, mucosal and unknown primary site melanomas accounted for 21% of the cases. Cutaneous melanomas included 33 nodular melanomas, 30 superficial spreading melanomas, 9 acral lentiginous melanomas, 1 lentigo maligna melanoma and 17 non-specified cutaneous melanomas.

**Table 1:** Clinicopathological features

Clinicopathological features	*N* (%)
**Age**^1^ median (range)	67 years (23–91)
**Gender**	
Female	59 (51.8%)
Male	55 (48.3%)
**Primary tumor**	
**Melanoma type**	
Cutaneous	90 (79.0%)
Ocular	11 (9.7%)
Mucosal	1 (0.9%)
Unknown primary site	12 (10.5%)
**Initial T**	
T1-2	32/96 (33.3%)
T3-4	64/96 (66.7%)
**Breslow's depth^2^** mean (±sd)	4.2 mm (±5.9)
**Initial N**	
N0	38/91 (41.8%)
N1-3	53/91 (58.2%)
**Initial M**	
M0	82/98 (83.7%)
M1	16/98 (16.3%)
**Mutation status**	
*BRAF* mutated	41/77 (53.6%)
*NRAS* mutated	13/35 (37.1%)
*cKIT* mutated	1/19 (5.3%)
**Metastatic lesion**	
**Clinical stage^1^**	
Stage III	41 (36.0%)
Stage IV	73 (64.0%)
**Biopsy sites**	
Lymph node	46 (40.4%)
Skin and subcutaneous tissue	24 (21.1%)
Lung	18 (15.8%)
Central Nervous System (CNS)	9 (7.9%)
Liver	8 (7.0%)
Gastrointestinal tract	4 (3.5%)
Other (bone, breast, peritoneum, thyroid and spleen)	5 (4.4%)
**Previous treatments**	
Treatment naïve	73/113 (64.6%)
Previously treated	40/113 (35.4%)
**Treatments received**	
Radiation therapy	27/40 (67.5%)
Chemotherapy	12/40 (30.0%)
Targeted therapy	11/40 (27.5%)
Immunotherapy	21/40 (52.5%)
**Follow-up**	**Mean (±sd)**
**Time from diagnosis to biopsy**	48.1 months (±60.6)
**Time from diagnosis to death/latest news**	66.3 months (±63.6)
**Time from biopsy to death/latest news**	17.6 months (±16.3)
**Deaths from any cause *N* (%)**	40 (35.1%)

1. At the time of sample collection. 2. Percentage based on the total number of patients with known Breslow's depth (*N* = 90). The 24 other patients had ocular, mucosal or unknown primary site melanoma.

Examined metastatic sites included lymph nodes (40%), skin and subcutaneous tissue (21%) and various viscera and central nervous system (39%). Besides surgery, 73 patients were treatment-naïve and 40 patients had received radiotherapy, chemotherapy, immunotherapy and/or targeted therapy. Of these, 10 patients had been treated by radiotherapy only. Twenty-one patients had received immunotherapy treatments including: immune checkpoint inhibitors (12 patients, of which 8 received anti-CTLA4 mAb, one received anti-PD1 mAb and 3 received both), cancer vaccines (3 patients), IFN-alpha (3 patients), cancer vaccines and IFN-alpha (1 patient) and anti-LAG-3 mAb (2 patients).

### Metastatic melanoma cells frequently express CD73

**Figure 1:**
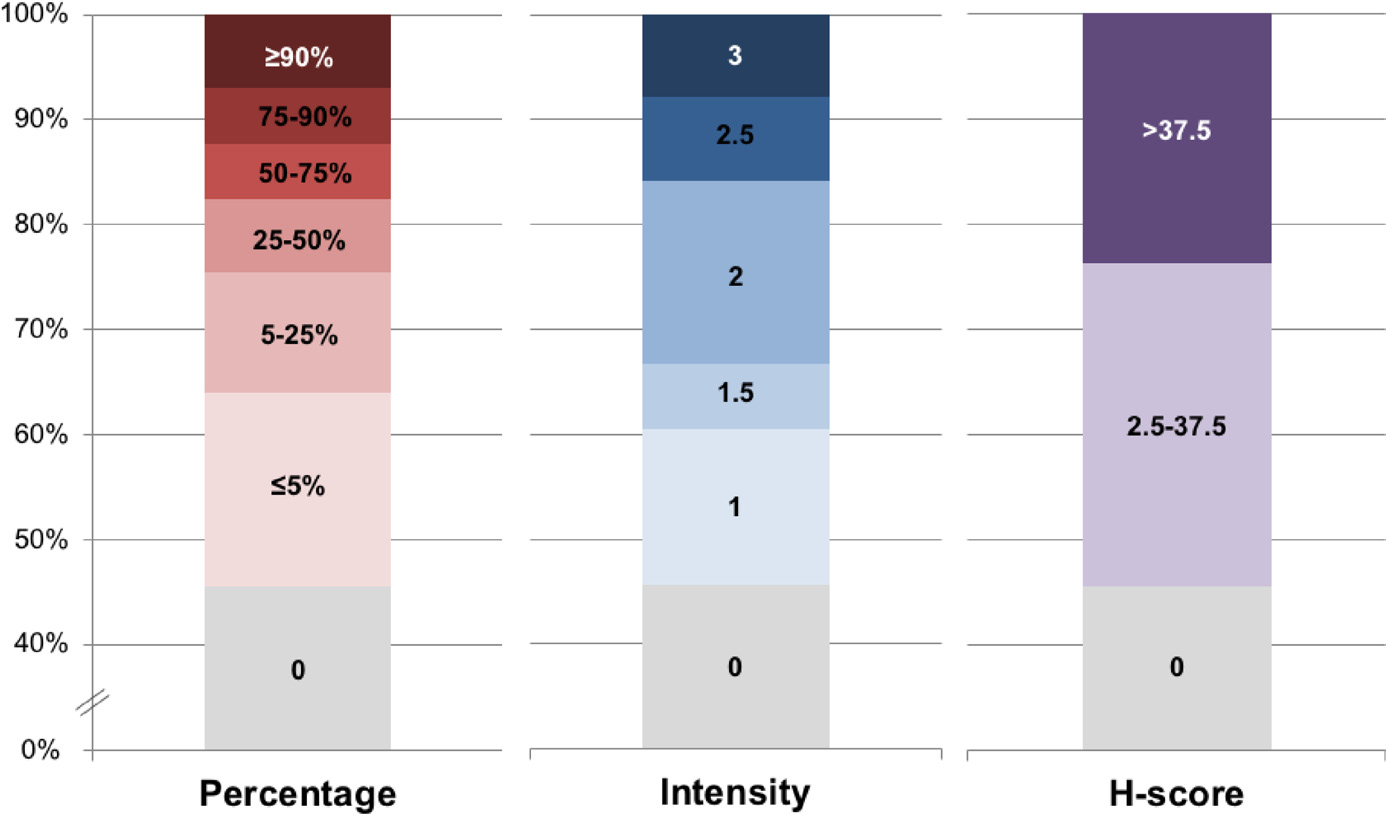
Distribution of CD73 staining in tumor cells Distribution of CD73 staining in the tumor cells of the 114 melanoma metastases according to percentage of TC staining (0 to >90%), intensity of staining (0 to 3) and H-score (0 to >37.5).

**Figure 2:**
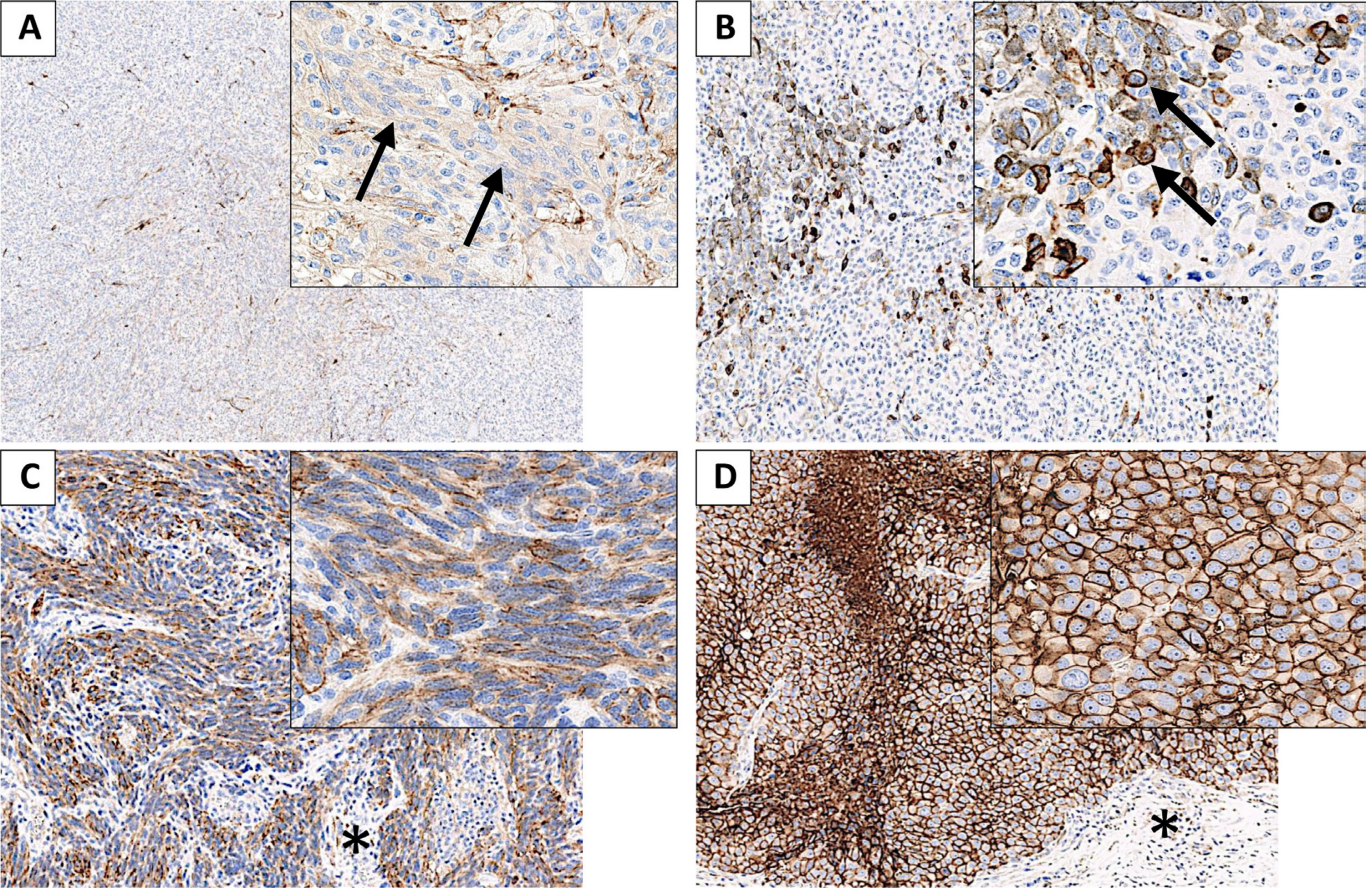
Metastatic melanoma lesions presenting CD73 staining in tumor cells (**A**) Melanoma metastasis presenting 5–25% of tumor cells (TC) staining (arrows), with an intensity of 1 and a cytoplasmic pattern. (**B**) Metastatic melanoma with ≤5% of TC staining, with moderate to strong intensity (averaged to 2.5) and a membrane pattern (arrows). (**C**) Metastatic melanoma with 75–90% of TC staining, intensity 2 to 3, mixed pattern (membrane and cytoplasmic staining). Surrounding stromal tissue is mostly unstained (^*^). (**D**) Metastatic melanoma with ≥90% of TC homogeneously staining, with strong intensity (3) and a membrane pattern. Surrounding stromal tissue is mostly unstained (^*^).

All lesions examined presented some expression of CD73 in endothelial or stromal cells which accounted for positive internal controls. Sixty-two samples (54%) contained CD73 positive tumor cells (TC) (Figure 1). Of these, 21 samples presented CD73 in less than 5% of TC, 21 in 5–50% of TC and 20 in 50–100% of TC (Supplementary Table 1). In seventeen metastases, TCs expressed CD73 with an intensity of 1, 27 with an intensity of 1.5–2 and 18 with an intensity of 2.5–3. H-scores varied from 0 to 285. Two thirds (39/62) stained with a membrane pattern, while 12 presented a cytoplasmic pattern and 11 presented both (Figure 2).

The association between CD73 expression in the metastases, the pathological features of the primary tumor and the clinical parameters was assessed by univariable multinomial regression analysis (Table 2). Age and gender did not significantly influence the H-score. Primary melanoma type and mutational status did not show any association with CD73 expression in metastatic lesions. Cases presenting deeper invasion (Breslow's depth) in the primary lesion presented significantly less CD73 in their metastases (*P* = 0.01). Patients with advanced pathologic stage (M1 and N1-3) at the time of the primary diagnosis tended to have more CD73 expression in their metastases. With respect to clinical stage at the time the metastasis was examined, lesions from patients at clinical stage IV tended to express more CD73 than those at clinical stage III.

**Table 2:** CD73 expression in tumor cells by H-score category and association with clinicopathological features

Variable	H-score 0 *N* (%)	H-score 2.5–37.5 *N* (%)	H-score > 37.5–285 *N* (%)	2.5–37.5 vs. 0 RRR (*P*-value)	>37.5–285 vs. 0 RRR (*P*-value)	Global *P*-value
**Age^1^** mean (±sd)	62.8 (±15.3)	63.2 (±14.9)	68.2 (±12.5)	1.00 (0.903)	1.02 (0.118)	0.241
**Gender**						0.482
Male (ref.)	22 (42.3%)	18 (51.4%)	15 (55.6%)	−	−	
Female	30 (57.7%)	17 (48.6%)	12 (44.4%)	0.69 (0.403)	0.59 (0.265)	
**Melanoma type^2^**						0.578
Cutaneous (ref.)	39 (75.0%)	29 (82.9%)	22 (81.5%)	−	−	
Mucosal	1 (1.9%)	0	0	4.59e–07 (0.993)	4.67e–07 (0.994)	
Ocular	6 (11.5%)	4 (11.4%)	1 (3.7%)	0.90 (0.874)	0.30 (0.273)	
Unknown primary site	6 (11.5%)	2 (5.7%)	4 (14.8%)	0.45 (0.347)	1.18 (0.811)	
**Initial T stage**						0.086
T1-2 (ref.)	9 (21.4%)	13 (41.9%)	10 (43.5%)	−	−	
T3-4	33 (78.6%)	18 (58.1%)	13 (56.5%)	0.38 (0.063)	0.35 (0.066)	
**Breslow's depth**	5.7 (±8.4)	2.6 (±1.7)	3.4 (±3.0)	0.77 (**0.023**)	0.89 (0.201)	**0.011**
mean (±sd)						
**Initial N stage**						0.762
N0 (ref.)	20 (45.5%)	10 (40.0%)	8 (36.4%)	−	−	
N1-3	24 (54.6%)	15 (60.0%)	14 (63.6%)	1.25 (0.661)	1.46 (0.482)	
**Initial M stage**						0.851
M0 (ref.)	40 (85.1%)	22 (84.6%)	20 (80.0%)	−	−	
M1	7 (14.9%)	4 (15.4%)	5 (20.0%)	1.04 (0.955)	1.43 (0.581)	
***BRAF* mutation**						0.741
No (ref.)	18 (48.7%)	11 (50.0%)	7 (38.9%)	−	−	
Yes	19 (51.4%)	11 (50.0%)	11 (61.1%)	0.95 (0.920)	1.49 (0.496)	
***NRAS* mutation**						0.755
No (ref.)	11 (68.8%)	6 (54.6%)	5 (62.5%)	−	−	
Yes	5 (31.3%)	5 (45.5%)	3 (37.5%)	1.83 (0.455)	1.32 (0.760)	
***cKIT* mutation**						0.567
No (ref.)	10 (90.9%)	4 (100.0%)	4 (100.0%)	−	−	
Yes	1 (9.1%)	0	0	2.67e−07 (0.996)	2.67e−07 (0.996)	
**Clinical stage^1^**						0.275
III (ref.)	22 (42.3%)	9 (25.7%)	10 (37.0%)	−	−	
IV	30 (57.7%)	26 (74.3%)	17 (63.0%)	2.12 (0.116)	1.25 (0.651)	
**Biopsy sites**						0.975
LN/skin/Sbc (ref.)	32 (61.5%)	21 (60.0%)	17 (63.0%)	−	−	
Lung	9 (17.3%)	6 (17.1%)	3 (11.1%)	1.02 (0.979)	0.63 (0.524)	
Central nervous system	3 (5.8%)	3 (8.6%)	3 (11.1%)	0.95 (0.939)	0.94 (0.929)	
Other	8 (15.4%)	5 (14.3%)	4 (14.8%)	1.52 (0.626)	1.88 (0.467)	
**Previous treatments**						**0.003**
None (ref.)	42 (80.8%)	17 (50.0%)	14 (51.9%)	−	−	
CT, RT, TT and/or IT	10 (19.2%)	17 (50.0%)	13 (48.2%)	4.2 (**0.003**)	3.9 (**0.009**)	
**TIMC quantity**						0.434
0 (ref.)	5 (9.6%)	1 (2.9%)	2 (7.4%)	−	−	
1, 2 or 3	47 (90.4%)	34 (97.1%)	25 (92.6%)	3.62 (0.250)	1.33 (0.744)	
**% of TIMC stained^3^**						0.872
0% (ref.)	31 (66.0%)	21 (61.76%)	17 (68.0%)	−	−	
1–50%^4^	16 (34.0%)	13 (38.2%)	8 (32.0%)	1.20 (0.698)	0.91 (0.861)	

Abbreviations: CT: chemotherapy; IT: immunotherapy; LN: lymph node; RRR: relative risk ratio (compared to ref.); RT: radiotherapy; Sbc: subcutaneous tissue; TIMC: tumor infiltrating mononuclear cells; TT: targeted therapy.

ref.: variable used as the reference for the statistical analysis.

The *P*-value was calculated by a univariable multinomial regression analysis. *P*-values < 0.05 are in bold.

1. At the time of sample collection. 2. Of the primary melanoma. 3. Of the samples containing TIMC (*n* = 106). 4. As no sample presented more than 50% of TIMC staining, categories above 50% are not represented.

Patients that received non-surgical treatment prior to sample collection had higher levels of CD73 in the metastases analyzed (*P* = 0.003). Only 14 of 73 treatment-naïve patients (19%) expressed CD73 with an H-score > 37.5. Conversely, 13 of 40 patients (33%) having previously received a non-surgical treatment (radiotherapy, chemotherapy, targeted therapy and/or immunotherapy) had a CD73 H-score > 37.5. More specifically, 33% (9/27) of patients treated with radiotherapy, 33% (4/12) of patients treated with chemotherapy, 36% (4/11) of patients treated with targeted therapy and 33% (7/21) of patients treated with immunotherapy (including 5/12, 42%, treated with immune checkpoint inhibitors) had a CD73 H-score > 37.5. Of the 8 patients who received an anti-CTLA4 monoclonal antibody (mAb), 6/8 presented some CD73 staining in TC (4/8 in > 25% of TC, 3/8 with an H-score > 37.5). Two of the 3 patients who received anti-CTLA4 plus anti-PD1 mAbs expressed CD73 in > 25% of TC (H-score > 37.5) and one was CD73-negative. The patient who received only an anti-PD1 mAb presented ≤5% of TC staining.

### Expression of CD73 in tumor infiltrating mononuclear cells (TIMC) correlates with a higher TIMC content

**Figure 3:**
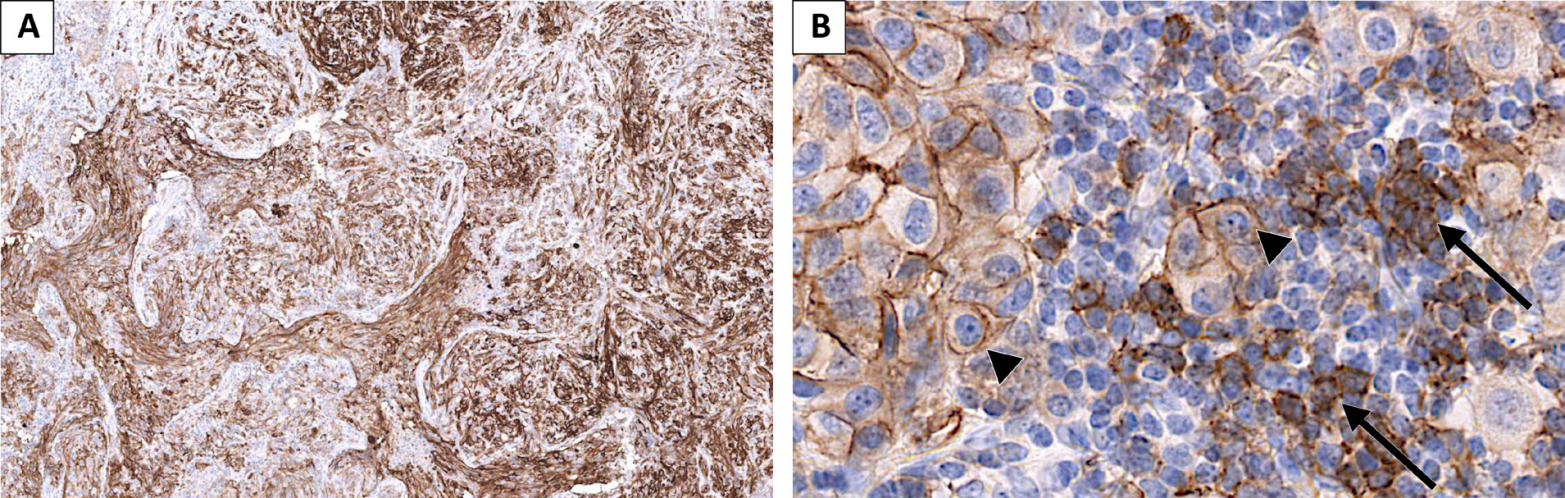
Melanoma lung metastasis presenting CD73 staining in tumor cells (TC) and in tumor infiltrating mononuclear cells (TIMC) (**A**) low magnification showing positive and negative areas and different intensities of staining; (**B**) TC (arrow heads) present membrane staining (75–90% of TC staining, intensity 2 to 3). A fraction of the TIMC present CD73 staining (arrows).

Most samples (93%) contained TIMC, mostly in low quantity (Table 3). Of these, 35% contained TIMC with some expression of CD73 (68% in ≤5% of TIMC and 32% in 5–50% of TIMC) (Figure 3, Table 3). Interestingly, specimens with more abundant TIMC were more likely to show CD73-positive TIMC (*P <* 0.001, Table 4). No correlation was found between CD73 expression in TIMC and in TC (Table 2).

**Table 3:** Non-adjusted survival analysis of quantity and staining of tumor infiltrating mononuclear cells

Variable	Deceased *N* (%)	Alive *N* (%)	OS from diagnosis HR (*P*-value)	OS from biopsy HR (*P*-value)
**TIMC quantity**			(0.271)	(0.428)
0 (ref.)	4 (10.0%)	4 (5.4%)	−	−
1	21 (52.5%)	37 (50.0%)	0.37	0.56
2	11 (27.5%)	24 (32.4%)	0.33	0.39
3	4 (10.0%)	9 (12.2%)	0.23	0.38
**TIMC quantity**				
0 (ref.)	4 (10.0%)	4 (5.4%)	−	−
1–3	36 (90.0%)	70 (94.6%)	0.34 (**0.047**)	0.48 (0.164)
**% of TIMC stained^1^**			(0.202)	(0.167)
0 (ref.)	29 (80.6%)	40 (57.1%)	−	−
1–5%	5 (13.9%)	20 (28.6%)	0.57	0.47
5–25%	2 (5.6%)	9 (12.9%)	0.30	0.37
25–50%^2^	0	1 (1.4%)	2.70e−14	4.72e−16
**% of TIMC stained^1^**				
0 (ref.)	29 (80.6%)	40 (57.1%)	−	−
1–50%^2^	7 (19.4%)	30 (42.9%)	0.46 (0.064)	0.43 (**0.044**)

Abbreviations: HR: Hazard ratio; OS: overall survival; TIMC: tumor infiltrating mononuclear cells.

1. Of the samples containing TIMC (*n* = 106). 2. As no sample presented more than 50% of TIMC staining, categories above 50% are not represented.

ref.: variable used as the reference for the statistical analysis.

The *P*-value was calculated by a Cox regression model. *P*-values < 0.05 are in bold.

**Table 4:** Tumor infiltrating mononuclear cells quantity and CD73 staining

TIMC quantity	*N* (%)	No TIMC staining	TIMC staining	*P*-value
0	8 (7.0%)	-	-	**0.00**
1	58 (50.9%)	47 (81.0%)	11 (19.0%)	
2	35 (30.7%)	17 (48.6%)	18 (51.4%)	
3	13 (11.4%)	5 (38.5%)	8 (61.5%)	

Abbreviations: TIMC: tumor infiltrating mononuclear cells.

The *P*-value was calculated by a Fisher's exact test. *P*-values < 0.05 are in bold.

### CD73 expression in metastatic melanoma cells correlates with poor clinical outcome

The average follow-up (from biopsy to date of death or latest news) was 17.75 months and 50% of the patients were followed for more than one year. By the end of the follow-up period, 40 patients had deceased from any cause (Table 1). Survival curves by initial T, N and M stage were as expected (Supplementary Figure 1) [20].

**Figure 4:**
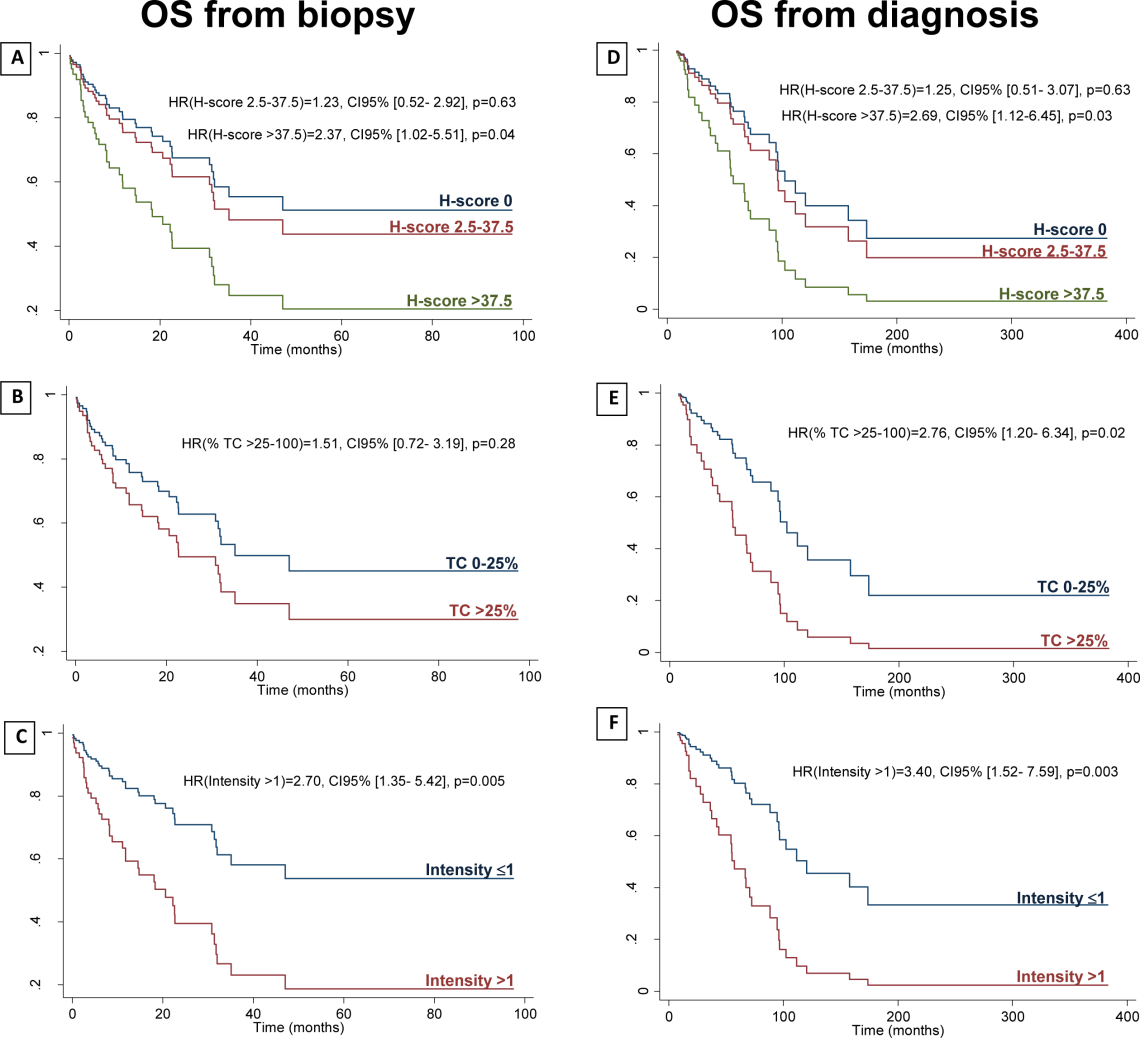
Adjusted overall survival from biopsy and from diagnosis OS from biopsy (**A**–**C**) and OS from diagnosis (**D**–**F**) by H-score (reference variable for the statistical analysis (ref.): H-score 0) (A, D); by percentage of TC staining (ref.: TC 0–25%) (B, E); and by intensity of staining (ref.: intensity ≤1) (C, F). HR: hazard ratio; OS: overall survival; TC: tumor cells. OS from biopsy is adjusted to initial T stage, previous treatments, and clinical stage at the moment of the biopsy (III vs. IV). OS from diagnosis is adjusted to initial T stage, previous treatments, and initial M stage.

To characterize the prognostic significance of CD73 expression, overall survival (OS) from biopsy was studied (Supplementary Table 2). In a multivariable analysis, initial T stage and previous treatments were found to be associated to the H-score at level of 20% (*P*-value < 0.20) (Supplementary Table 3). As clinical stages III and IV have very different prognosis, this variable was used to adjust OS from biopsy, together with initial T stage and previous treatments [20]. When adjusting to these variables, higher levels of CD73 expression in TC correlated with decreased OS from biopsy: a) H-score > 37.5 vs. H-score 0: Hazard ratio (HR) 2.37, *P* = 0.04; b) %TC > 25% vs. %TC 0–25%: HR 1.51, *P* = 0.28; c) intensity > 1 vs. intensity ≤1: HR 2.70; *P* = 0.005) (Figure 4). Concordantly, CD73 expression in TC also correlated with decreased OS from diagnosis (Figure 4).

Regarding TIMC quantity, the presence of TIMC (1–3 vs. 0) associated with improved OS from diagnosis (Table 3). In a non-adjusted analysis, CD73 expression in TIMC (1–50% vs. 0%) significantly improved OS from biopsy (HR = 0.43, *P* = 0.044) (Table 3).

### CD73 expression features temporal and spatial heterogeneity

Multiple metastatic lesions in 16 patients were analyzed to gain insight into CD73 expression heterogeneity (Supplementary Table 4). Sequential biopsies of the same lesion at ≥2 months interval showed either stability (three cases fully concordant and one case differing by <5%) or an increase (three cases) in the percentage of TC staining over time. Metastatic lesions in different locations biopsied at the same time showed approximately the same percentage of TC staining (five cases fully concordant and two cases differing by < 25%). Finally, different metastatic lesions biopsied at different times showed either an increase (three cases), stability (two cases differing by < 5%) or a decrease (one case) in the percentage of TC staining.

## DISCUSSION

This study shows that all metastatic melanomas presented some CD73 expression either by endothelial, stromal, tumoral or immune cells. Fifty-four percent presented CD73 expression in TC (of those, 52% in > 25% of TC and 70% with an intensity of 2 or more) and approximately a third of the samples containing TIMC had CD73-positive TIMC.

We found that CD73 expression in metastatic melanomas was significantly associated with a lower Breslow's depth of the primary lesion. Interestingly, a recent study analyzed CD73 expression by IHC in human primary melanomas (*n* = 126) and cutaneous metastases (*n* = 70). CD73 expression in primary melanomas (119/126) significantly associated with increased tumor thickness, ulceration and positive sentinel lymph node, while no significant correlation with survival was found. Almost all cutaneous melanoma metastasis expressed CD73 (69/70) but no correlation was found with age, gender or necrosis [21]. The discrepancy between these and our observations may be explained by the methodology. In particular, we assessed CD73 in metastatic lesions only, and our cohort is biased towards a subset of melanoma patients who subsequently develop metastatic melanoma, meaning that among the cases with lower Breslow's depth only the minority with an unfavorable outcome was selected. Consequently, this result should be cautiously interpreted, and it is also possible that the biological relevance of CD73 expression in melanoma varies according to the clinical setting in patients with localized versus metastatic disease.

Non-surgical treatments prior to biopsy were also significantly associated with a higher CD73 expression in TC. Specifically, 42% of patients treated with immune checkpoint inhibitors presented an H-score > 37.5 versus 33% of patients having previously received any non-surgical treatment. Despite the small numbers (only 12 patients treated with immune checkpoint inhibitors), this observation is consistent with a recent article. describing an upregulation of CD73 expression in melanoma TC in patients progressing under adoptive T cell transfer or immune checkpoint blockade [21]. This observation supports the rational for combining current immunotherapy strategies with antibody-mediated CD73 blockade. In this context, evaluating CD73 expression before and after current standard immune checkpoint treatments would be relevant.

The analysis of multiple metastatic lesions from the same patient revealed that CD73 expression is not always stable. This analysis seemed to indicate that the location of the metastasis does not greatly influence CD73 expression, whereas time and/or treatment might increase its expression. It would be of interest to study paired primary and metastatic lesions to clarify the association between CD73 expression and the invasive capacity of the primary tumor (Breslow's depth) and to gain insight about the dynamics of CD73 expression.

Higher quantities of TIMC infiltrating tumors were associated with higher proportions of TIMC expressing CD73, however, the evaluation of the proportion of immune cells stained was challenging due to the frequent colocalization of immune cells with stroma and vessels. IHC staining with anti-CD3 and anti-CD20 antibodies was performed in three lung metastases. In these samples, CD73 staining colocalized with CD20 staining (Supplementary Figure 2). Further studies would be useful to better characterize the immune cell subtypes expressing CD73 in the melanoma milieu.

Higher levels of CD73 expression in TC were significantly associated with decreased OS from biopsy. This result supports the notion that CD73 expression in TC of metastatic lesions is a factor of poor prognosis for melanoma patients. Contrariwise, CD73 expression in TIMC was significantly associated with improved OS. Notably, higher CD73 expression in TIMC correlated with higher TIMC quantity. An explanation for the improved OS would be that the well-known benefit of higher tumor lymphocytic infiltration might be greater than the deleterious effect of increasing CD73 activity in the tumor microenvironment. Other two factors worth considering would be the participation of CD73 in lymphocyte trafficking to the tumor microenvironment [6] and the better prognosis associated with B-cell infiltration in melanomas (Gourdin N. *et al*. submitted). Still, this needs to be interpreted with caution due to the difficulty in assessing the proportion of TIMC staining. This apparent double-edged sword character of CD73 expression in the tumor microenvironment should be taken into account when considering anti-CD73 targeting strategies.

Noteworthy, in mouse models, targeting CD73 synergized with anti-PD-1 and anti-CTLA4 mAbs, suggesting that these might be promising combinations [22]. CD73's potential as a therapeutic target led to a phase I trial with an anti-CD73 mAb alone or in combination with an anti-PD-L1 mAb (NCT02503774) in advanced solid tumors [23].

To our knowledge, this is the first study to characterize CD73 expression in human metastatic melanomas without restriction to a specific metastatic site, also, it is the first to characterize CD73 expression in melanoma TIMC. Despite its weaknesses (relatively small sample size, only evaluating metastatic lesions and a heterogeneous population), this study helps to characterize CD73 expression in TC and TIMC, bringing insight about the relation of CD73 expression with clinicopathological characteristics, treatment and prognosis. This study supports CD73 as a frequent and influent molecule in metastatic melanoma. Also, it encourages the study of anti-CD73 therapies in this population.

## MATERIALS AND METHODS

### Tissue samples and clinical data

Formalin-fixed paraffin-embedded metastatic melanoma samples collected between 2005 and 2016 were retrieved from the archives of the Institute of Pathology of the Lausanne University Hospital. Overall, 713 samples were identified, from 560 patients. Of those, 114 patients had samples suitable for the purpose of this project (surgical specimens or needle biopsies of sufficient size and fulfilling ethical requirements). For each patient, the first eligible metastatic lesion was selected. Sixteen patients had more than one eligible lesion, resulting in 25 extra samples which were examined in a supplementary analysis. Clinical and pathological data were obtained from medical records.

The study protocol was approved by the cantonal ethics committee on human research (Lausanne) (protocol 17/15). All samples were used in accordance with the Declaration of Helsinki.

### Immunohistochemistry

IHC was performed using a CD73-specific antibody (D7F9A, rabbit monoclonal, #13160, Cell Signaling), using the Ventana BenchMark automated stainer. Briefly, deparaffinized slides were pre-treated with CC1 for 60 minutes and incubated with the anti-CD73 primary antibody for 60 minutes at 37° C (dilution 1:100). The Ultraview DAB detection kit (ref. 760–500) was used, followed by hematoxylin counterstaining. An external control (reactive tonsil) was stained in each batch (Supplementary Figure 3).

An adjacent section was stained with hematoxylin and eosin (H&E) (Ventana HE 600 system) for morphological reappraisal and to assist IHC interpretation.

IHC slides were scanned using NanoZoomer NDP 1.0 slide scanner. Leica Biosystems SlidePath software (version 4.0.7) was used for visualization and image capture.

### Morphological and immunohistochemistry analysis

Using the H&E slide, the quantity of TIMC (0: none; 1: scarce; 2: moderate; 3: abundant) was reported (Supplementary Figure 4).

On IHC stainings, CD73 expression was evaluated in TC and TIMC. For TC, the staining pattern (membrane, cytoplasmic or both), the percentage expressing CD73 (percentage groups: 0, ≤5%, 5–25%, 25–50%, 50–75%, 75–90% or ≥90%) and the intensity of staining (0, 1, 2 or 3) were reported. When intensity was significantly heterogeneous, an average value between the highest and lowest intensities observed was taken (1.5, 2 or 2.5). The H-score was calculated as percentage (mean value of each percentage group) x intensity (H-score: 0–285). For TIMC, the percentage staining was assessed (0%, ≤5%, 5–25%, 25–50%, 50–75% or ≥75%). Staining in endothelium and stroma was used as positive internal controls. Evaluation of slides was performed independently by a pathology resident (IM) and reviewed together with a senior pathologist (LdL) to reach a consensus.

### Statistical analysis

Statistical analysis was performed using Stata 14 software (StataCorp. 2015. Stata Statistical Software: Release 14. College Station, TX: StataCorp LP). The H-score was categorized into three groups: none (H-score = 0), moderate (H-score = [2.5–37.5]) and high (H-score > 37.5). The division between the last two groups corresponds to the percentile 75. Categorical data were summarized by frequencies and percentages, and continuous variables by their mean (± standard deviation). The associations between the explanatory factors and H-score were then assessed using univariable multinomial logistic regression models. The strength of the association was measured by the Relative Risk Ratio (RRR) and calculated *P*-values. Factors which were associated to the H-score at level of 20% (*P*-value < 0.20) were considered in a backward procedure to fit a multivariable model. These factors were used as adjusting variables to assess the association of the H-score with OS. For this last analysis, a Cox proportional-hazards regression was performed.

## SUPPLEMENTARY MATERIALS FIGURES AND TABLES



## Abbreviations

H&E: hematoxylin and eosin
IHC: immunohistochemistry
mAb(s): monoclonal antibody(ies)
OS: overall survival
RRR: relative risk ratio
TC: tumor cells
TIMC: tumor infiltrating mononuclear cells
