# Supplementary material for: CD73 expression and clinical significance in human metastatic melanoma
Source: Oncotarget. 2018 Jun 1;9(42):26659–69. doi: 10.18632/oncotarget.25426 (PMC6003551; doi:10.18632/oncotarget.25426)
Supplement: Supplementary file 1 [file oncotarget-09-26659-s001.pdf]

CD73 expression and clinical significance in human metastatic melanoma

SUPPLEMENTARY MATERIALS

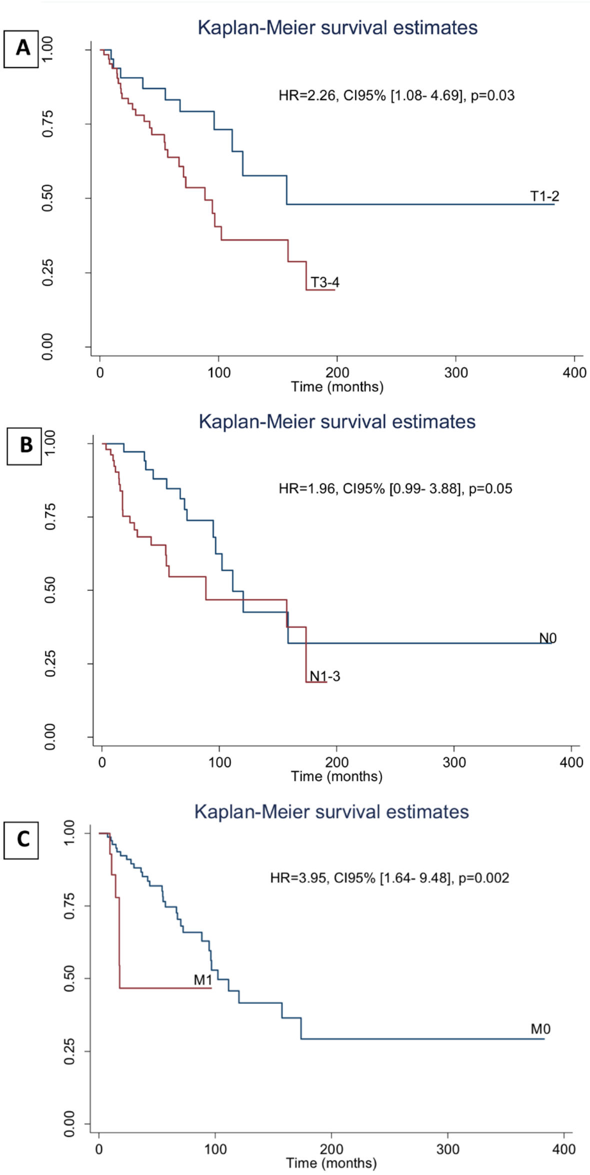

Supplementary Figure 1: Overall survival by initial T, N and M stage. (**A**) by initial T stage (reference variable for the statistical analysis (ref.): T1-2); (**B**) by initial N stage (ref.: N0); (**C**) by initial M stage (ref.: M0). HR: hazards ratio. The *P*-value was calculated by a Cox regression model.

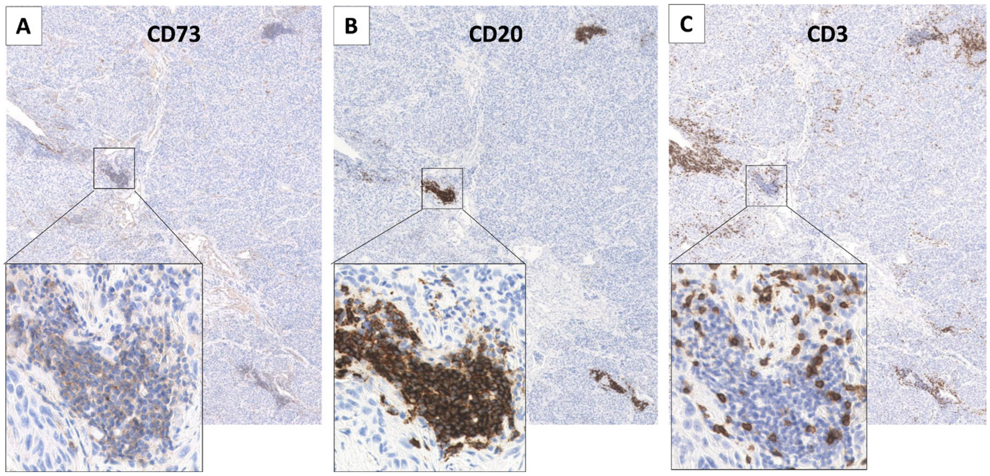

Supplementary Figure 2: CD73, CD20 and CD3 expression in a melanoma lung metastasis. Images A, B and C represent adjacent sections of the same melanoma lung metastasis stained with CD73, CD20 and CD3-specific antibodies, respectively. Tumor cells, occupying the majority of the image, are not immunostained (in blue). Tumor infiltrating mononuclear cells are disposed in small niches and present some CD73 (**A**), CD20 (**B**) and CD3 (**C**) staining (in brown). Note that CD73 staining colocalizes better with CD20 staining than with CD3 staining.

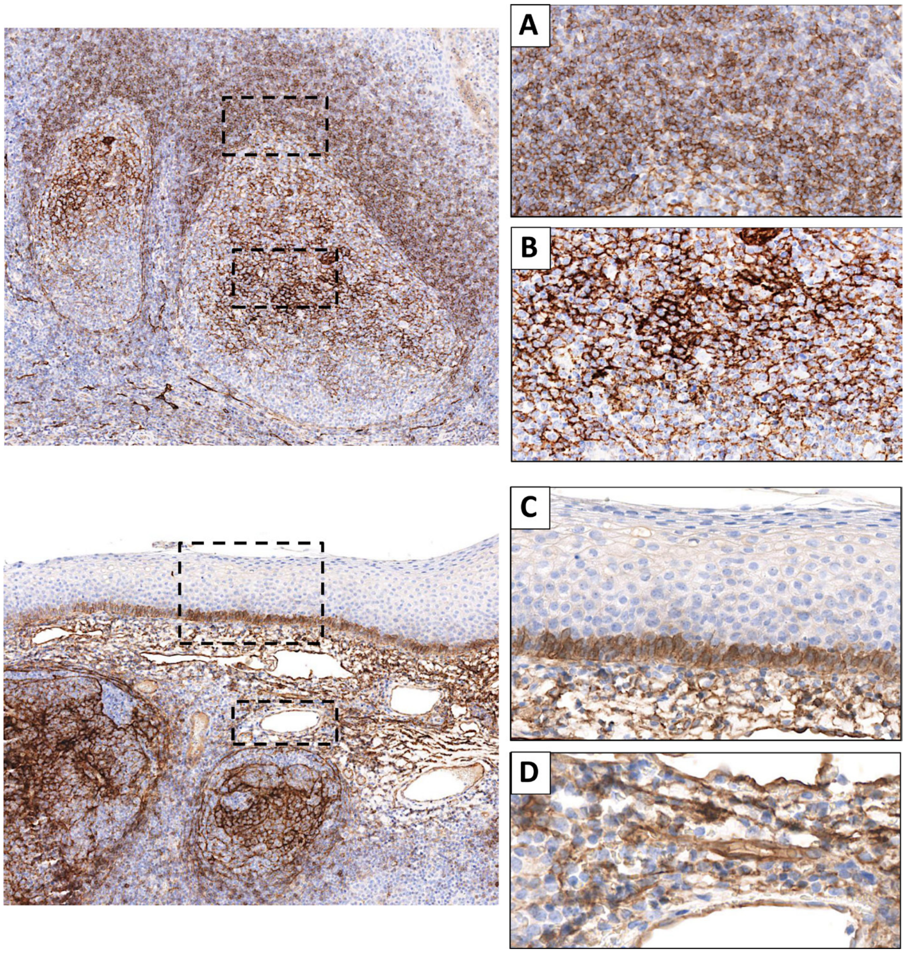

Supplementary Figure 3: External control-Tonsil. Staining was observed in lymphocytes of the mantle zone (with a membrane pattern - **A**), in germinal centers (with a follicular dendritic cell pattern - **B**), in the basal cell layer of the epithelium (**C**), in vascular and lymphatic endothelial cells and in stromal cells (**D**).

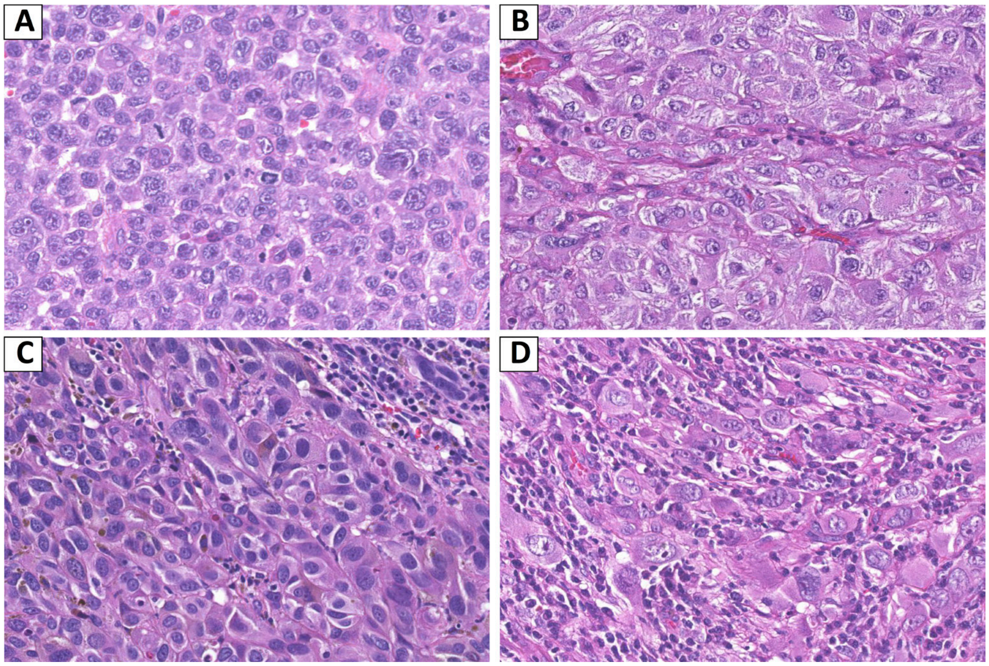

Supplementary Figure 4: Quantification of tumor infiltrating mononuclear cells. Samples showing none (quantity = 0- **A**) (Note: the immune cells present in this example are polymorphonuclear leukocytes), scarce (quantity = 1 - **B**), moderate (quantity = 2 - **C**) and abundant (quantity = 3 - **D**) tumor infiltrating mononuclear cells (TIMC). For lymph node metastases, only TIMC in between the tumor cells were considered, while in all other sites both intra and peritumoral TIMC were taken into account.

Supplementary Table 1: Staining distribution of tumor cells

Variable	*N* (%)
**Tumor cells**	
**Percentage of TC staining**	
0%	52 (45.6%)
≤5%	21 (18.4%)
5–25%	13 (11.4%)
25–50%	8 (7.0%)
50–75%	6 (5.3%)
75–90%	6 (5.3%)
≥90%	8 (7.0%)
**Intensity of TC staining**	
0	52 (45.6%)
1	17 (14.9%)
1.5	7 (6.1%)
2	20 (17.5%)
2.5	9 (7.9%)
3	9 (7.9%)
**H-score**	
0	52 (45.6%)
2.5–37.5	35 (30.7%)
>37.5–285	27 (23.7%)

Abbreviations: TC: tumor cells.

Supplementary Table 2: Non-adjusted survival analysis of CD73 staining in tumor cells

Variable	Deceased *N* (%)	Alive *N* (%)	OS from diagnosis HR (*P*-value)	OS from biopsy HR (*P*-value)
**% of TC stained**			(**0.008**)	(**0.001**)
0 (ref.)	15 (37.5%)	37 (50.0%)	-	-
≤5%	10 (25.0%)	11 (14.9%)	1.37	2.54
5–25%	1 (2.5%)	12 (16.2%)	0.20	0.26
25–50%	4 (10.0%)	4 (5.4%)	2.59	3.21
50–75%	4 (10.0%)	2 (2.7%)	2.89	4.28
75–90%	0	6 (8.1%)	2.04e-16	4.05e-15
≥90%	6 (15.0%)	2 (2.7%)	2.40	3.33
**% of TC stained**				
0–25% (ref.)	26 (65.0%)	60 (81.1%)	-	-
>25%	14 (35.0%)	14 (18.9%)	2.15 (**0.022**)	2.03 (**0.033**)
**Intensity of staining**			(0.102)	(**0.001**)
0 (ref.)	15 (37.5%)	37 (50.0%)	-	-
1	3 (7.5%)	14 (18.9%)	0.47	0.57
1.5	1 (2.5%)	6 (8.1%)	0.73	0.82
2	13 (32.5%)	7 (9.5%)	2.30	5.49
2.5	3 (7.5%)	6 (8.1%)	1.44	1.23
3	5 (12.5%)	4 (5.4%)	1.36	2.19
**Intensity of staining**				
0–1 (ref.)	18 (45.0%)	51 (68.9%)	-	-
1.5–3	22 (55.0%)	23 (31.1%)	2.05 (**0.024**)	2.91 (**0.001**)
**H-score**			(0.080)	(**0.045**)
0 (ref.)	15 (37.5%)	37 (50.0%)	-	-
2.5–37.5	11 (27.5%)	24 (32.4%)	0.88	1.33
>37.5	14 (35.0%)	13 (17.6%)	2.06	2.59

Abbreviations: HR: Hazard ratio; OS: overall survival; TC: tumor cells.
ref.: variable used as the reference for the statistical analysis.
The *P*-value was calculated by a Cox regression model. *P*-values < 0.05 are in bold.

Supplementary Table 3: Multivariable analysis of H-score

Variable	RRR	*P*-value	Global *P*-value
**H-score 0 (ref.)**	-	-	**0.005**
**H-score 2.5–37.5**			
T3-4	0.38	0.082	
Previous treatment	4.56	**0.007**	
**H-score >37.5–285**			
T3-4	0.33	0.060	
Previous treatment	4.81	**0.009**	

Initial T stage and previous treatments were the variables found to be associated to the H-score at level of 20% (*P*-value < 0.20).
Abbreviations: RRR: relative risk ratio.
The *P*-value was calculated by a multinomial logistic regression model. *P*-values < 0.05 are in bold.

Supplementary Table 4: Heterogeneity of CD73 expression in multiple melanoma metastases

Patient<br>Primary melanoma subtype	Time from diagnosis<br>(previous treatment)	First sample collection	Time[1]<br>(treatment[2])	Second sample collection	Time[1]<br>(treatment[2])	Third sample collection
**Patient 1**<br>Cutaneous	16 months<br>(RT)	**Right lung**<br>(pleural invasion)<br>TC 75–90%<br>TIMC 0	11 months<br>(anti-CTLA4)	**Right pleura**<br>TC ≥90%<br>TIMC 0	–	–
**Patient 2**<br>Cutaneous	3 months<br>(None)	**SCT (left inguinal)**<br>TC ≤5%<br>TIMC 0	7 months<br>(None)	**Left inguinal LN**<br>TC 0<br>TIMC 0	–	–
**Patient 3**<br>Cutaneous	62 months<br>(None)	**Skin (right breast)**<br>TC 0<br>TIMC 0	6 months<br>(None)	**Skin (right breast)**<br>TC 0<br>TIMC 0	3 months<br>(None)	**Skin (right breast)**<br>TC 0<br>TIMC 0<br>**Skin (right hip)**<br>TC 0<br>TIMC 0<br>**Right thorax LN**<br>TC 0<br>TIMC 0
**Patient 4**<br>Cutaneous	34 months<br>(None)	**Skin (left breast)**<br>TC 0<br>TIMC 0	3 months<br>(None)	**Skin (left breast)**<br>TC 0<br>TIMC ≤5%	2 months<br>(None)	**Skin (left breast)**<br>TC 0<br>TIMC 0
**Patient 5**<br>Cutaneous	24 months<br>(None)	**Left axillary LN**<br>(effraction)<br>TC 5–25%<br>TIMC 0<br>**Skin (back)**<br>TC 0<br>TIMC 5–25%	7 months<br>(None)	**Skin (left axillary)**<br>TC 25–50%<br>TIMC 5–25%	–	–
**Patient 6**<br>Cutaneous	64 months<br>(None)	**Left tibia SCT**<br>TC 0<br>TIMC ≤5%	38 months<br>(None)	**Left tibia SCT**<br>TC 0<br>TIMC 0	–	–
**Patient 7**<br>Cutaneous	45 months<br>(CT, RT, dabrafenib, anti-CTLA4)	**Skin (jaw)**<br>TC ≤5%<br>TIMC 0	8 months<br>(CT, dabrafenib, trametinib)	**Autopsy**<br>**Skin (jaw)**<br>TC ≥90%<br>TIMC 0<br>**Lung**<br>TC 75–90%<br>TIMC 0<br>**Heart**<br>TC ≥90%<br>TIMC 0	–	–
**Patient 8**<br>Unknown primary site	0 months<br>(None)	**Skin (frontal)**<br>TC 0<br>TIMC ≤5%<br>**Left inguinal LN**<br>TC 0<br>TIMC 0	–	–	–	–
**Patient 9**<br>Cutaneous	24 months<br>(None)	**Skin (right ankle)**<br>TC 0<br>TIMC 0<br>**Right inguinal LN**<br>TC 0<br>TIMC 0	22 months<br>(RT, CT)	**Brain**<br>TC 0<br>TIMC 0	–	–

**Patient 10**<br>Cutaneous	144 months<br>(CT, Melan-A, INF-alpha)	**Lung**<br>TC ≤5%<br>TIMC 0<br>**Left flank LN**<br>TC 0<br>TIMC 0	–	–	–	–
**Patient 11**<br>Cutaneous	88 months<br>(CT, INF-alpha)	**Skin (left inguinal)**<br>TC 0<br>TIMC 0<br>**Aortic LN**<br>TC ≤5%<br>TIMC 0	–	–	–	–
**Patient 12**<br>Cutaneous	5 months<br>(None)	**Left axillary LN**<br>TC 0<br>TIMC ≤5%	19 months<br>(Anti-CTLA4)	**Intestine**<br>TC 75–90%<br>TIMC ≤5%	–	–
**Patient 13**<br>Unknown primary site	1 month<br>(None)	**Right inguinal LN**<br>TC 0<br>TIMC ≤5%	19 months<br>(RT)	**Breast**<br>TC ≤5%<br>TIMC 25–50%	–	–
**Patient 14**<br>Ocular	20 months<br>(RT, bevacizumab)	**Liver**<br>TC 0<br>TIMC 0	14 months<br>(RT, anti-CTLA4)	**Intestine**<br>TC 25–50%<br>TIMC 0	–	–
**Patient 15**<br>Cutaneous	110 months<br>(RT, CT, anti-CTLA4, anti-PD1)	**Left inguinal LN**<br>TC 0<br>TIMC 0	6 months<br>(None)	**Skin (right thigh)**<br>TC 25–50%<br>TIMC 0	–	–
**Patient 16**<br>Unknown primary site	13 months<br>(Anti-CTLA4)	**Skin (right torso)**<br>TC 50–75%<br>TIMC 5–25%	23 months<br>(RT, CT, anti-CTLA4, anti-PD, dabrafenib, trametinib)	**Skin (right thorax)**<br>TC 0<br>TIMC 5–25%	–	–

Abbreviations: CT: chemotherapy; LN: lymph node; RT: radiotherapy; SCT: subcutaneous tissue.
1. Time between the two sample collections. 2. Treatment received between the two sample collections.
Patients 1-7: same lesion biopsied at different times. Patients 3, 5, 7-11: different lesions biopsied at the same time (or less than 1 month apart). Patients 9, 12-16: different lesions biopsied at different times.
